# Executive functioning and treatment outcome among adolescents undergoing cognitive‐behavioral therapy for binge‐eating disorder

**DOI:** 10.1111/jcpp.14031

**Published:** 2024-06-28

**Authors:** Andrea B. Goldschmidt, Kwonho Jeong, Lan Yu, Amy H. Egbert, Ricarda Schmidt, Anja Hilbert

**Affiliations:** ^1^ Department of Psychiatry University of Pittsburgh School of Medicine Pittsburgh PA USA; ^2^ Department of Medicine University of Pittsburgh School of Medicine Pittsburgh PA USA; ^3^ Department of Psychological Sciences University of Connecticut Storz CT USA; ^4^ Department of Psychosomatic Medicine and Psychotherapy, Behavioral Medicine Research Unit, Integrated Research and Treatment Center AdiposityDiseases Leipzig University Medical Center Leipzig Germany

**Keywords:** Executive functioning, binge eating, loss of control eating, adolescent, cognitive‐behavioral therapy

## Abstract

**Background:**

Cognitive‐behavioral therapy (CBT) is an evidence‐supported treatment for adolescents with binge‐eating disorder (BED). Executive dysfunctions, which are associated with binge eating and elevated body weight in youth, may undermine CBT outcomes by making it difficult for youth to engage with or adhere to treatment, including recalling and/or implementing intervention strategies in real‐world contexts.

**Methods:**

We assessed 73 adolescents [82.2% female; *M*_age_ = 15.0 ± 2.5 year; *M* baseline standardized body mass index (zBMI) = 1.9 ± 1.0 kg/m^2^] with BED at baseline, posttreatment, 6‐, 12‐, and 24‐month follow‐up. Linear mixed models examined the effects of baseline executive functioning (EF) on loss of control (LOC) eating and weight change following CBT. Linear and logistic regressions probed associations between EF, attendance, and attrition.

**Results:**

More impulsive decision‐making, as reflected in higher baseline scores on the Iowa Gambling Task, predicted better attendance (*β* = .07; *p* = .019) and more frequent LOC eating following treatment (*β* = .12; *p* = .017). Lower cognitive flexibility, as reflected in lower baseline *T*‐scores on the Comprehensive Trail Making Test complex sequencing index, predicted higher zBMI following treatment (*β* = −.03; *p* = .003). Inhibition, concentration, attention, and parent‐reported EF behavior symptoms were not associated with outcome, attendance, or attrition.

**Conclusions:**

More impulsive decision‐making and lower cognitive flexibility were associated with suboptimal response to CBT for BED, although findings should be interpreted with caution in light of the sample size and waitlist control design. Future research should examine whether strengthening EF could improve eating and weight outcomes among adolescents with BED who have lower pre‐treatment EF.

Loss of control (LOC) eating, involving a sense that one cannot control what or how much one is eating (e.g. American Psychiatric Association, 2013), affects 10%–15% of young people in community samples (Goldschmidt et al., 2015; Schlüter, Schmidt, Kittel, Tetzlaff, & Hilbert, 2016) and >30% of youth with overweight/obesity (He, Cai, & Fan, 2016). LOC eating is associated with adverse physical and psychosocial health consequences, including excess weight status, eating‐related and general psychopathology, impairments in quality of life, and interpersonal dysfunction, independent of episode size and frequency (Goldschmidt, 2017; Tanofsky‐Kraff, Schvey, & Grilo, 2020). Yet, only ~2% of youth meet criteria for binge‐eating disorder (BED) per Diagnostic and Statistical Manual of Mental Disorders, fifth edition (DSM‐5; American Psychiatric Association, 2013) criteria (Silén & Keski‐Rahkonen, 2022), which include recurrent objectively large binge‐eating episodes (≥1 episode per week, on average, for 3 months) in the absence of regular use of compensatory behaviors. Some researchers have proposed adapting the criteria for BED to allow for a more flexible and age‐appropriate definition of binge eating (i.e. LOC while eating, regardless of episode size and/or at a lower frequency threshold; Bravender et al., 2010), which is reflected in the recently updated International Classification of Diseases (ICD‐11) taxonomy (World Health Organization, 2019).

In addition to cross‐sectional and prospective associations with excess weight status and psychosocial distress (Hilbert, Hartmann, Czaja, & Schoebi, 2013; Tanofsky‐Kraff et al., 2006, 2011), LOC eating in youth is associated with relative deficits in neurocognitive functioning, particularly within the executive functioning domain (i.e. cognitive self‐regulation processes that govern goal‐directed behavior; Lavagnino, Arnone, Cao, Soares, & Selvaraj, 2016), including working memory, planning (Goldschmidt et al., 2018), and inhibitory control (Kittel, Schmidt, & Hilbert, 2017). These relative deficits may facilitate binge eating or overeating (Schmidt, Wandrer, Boutelle, Kiess, & Hilbert, 2023), thereby exacerbating the risk of excess weight gain (Goldschmidt, Hipwell, Stepp, McTigue, & Keenan, 2015) and undermining weight control attempts (Naets, Vervoort, Verbeken, & Braet, 2018) by, for example, diminishing attempts to create and implement a healthy meal plan or inhibiting goal‐incompatible responses to food cues (Eichen, Pasquale, Twamley, & Boutelle, 2021). The effects of executive functioning on psychological treatment outcomes for LOC eating in youth are currently unknown.

Cognitive‐behavioral therapy (CBT) is a first‐line treatment for adult BED (Hilbert et al., 2019), with effects on binge eating surpassing waitlist control and credible control conditions in some, but not all, trials (Linardon, Wade, de la Piedad Garcia, & Brennan, 2017). Accumulating data suggest that it is also an efficacious treatment for adolescents, outperforming delayed treatment in youth with recurrent binge eating (DeBar et al., 2013) and BED (Hilbert, Petroff, Neuhaus, & Schmidt, 2020), while comparative efficacy relative to credible control treatment is currently unknown. CBT is predicated on the restraint model of binge eating (Polivy & Herman, 1985), which presupposes that binge eating results from perceived lapses in overly strict and inflexible attempts to regulate one's eating (e.g. breaking a dietary rule) driven by an overly inflated emphasis on shape and weight in one's scheme for self‐evaluation (Fairburn, 2008). As such, CBT focuses on normalizing eating behavior and maladaptive cognitions about eating, shape and weight via interventions such as self‐monitoring, stimulus control, and cognitive restructuring (Beck, 2011). Although CBT for BED does not directly target weight *loss*, treatment targets include healthy regulation of eating and physical activity (Hilbert, 2013) to support stabilization of BMI and/or modest BMI reductions for adolescents who are still growing. Further, because LOC and binge eating are prospectively associated with excess weight gain and adiposity in both adults (Barnes, Blomquist, & Grilo, 2011; Blomquist et al., 2011) and some (Tanofsky‐Kraff et al., 2006, 2009), but not all (Hilbert et al., 2013; Hilbert & Brauhardt, 2014), studies of adolescents, weight change is often considered as a secondary outcome in CBT clinical trials (Palavras, Hay, Filho, & Claudino, 2017).

Importantly, CBT models assume that patients have basic executive functioning skills to appropriately recall and implement treatment techniques in the contexts/moments when they are needed, while inadequately considering that self‐regulation capacity (i.e. ability to override undesirable impulses in pursuit of longer‐term goals; Johnson, Pratt, & Wardle, 2012) may be inherently lower in some youth (Lavagnino et al., 2016), and remains in flux through adolescent development (Best & Miller, 2010; Ferguson, Brunsdon, & Bradford, 2021) underscored by normative changes in neurobiology (Casey, 2015). This may make it challenging for some adolescents to implement skills learned during treatment that largely depend on self‐control in the moments and contexts they are needed most (e.g. remembering to employ stimulus control to prevent binge eating or overeating in high‐risk locations). While the effects of executive functioning on CBT outcome for LOC eating are unknown, some (but not all; Dingemans, van Son, Vanhaelen, & van Furth, 2020; Godovich et al., 2020; Hybel, Mortensen, Lambek, Højgaard, & Thomsen, 2017) data suggest that poorer executive functioning may predict or moderate treatment outcomes for related mental health conditions in children (Flessner et al., 2010; McNamara et al., 2014) and adults (including eating disorders; Lucas et al., 2021).

The purpose of this study was to examine the effects of pre‐treatment executive functioning on treatment outcomes in the context of CBT for adolescents with BED. We hypothesized that those with poorer functioning at pre‐treatment would show attenuated improvements in LOC eating and weight at posttreatment and 6‐, 12‐, and 24‐month follow‐up. An exploratory aim was to additionally examine the impact of pre‐treatment executive functioning on treatment engagement (i.e. attendance, attrition).

## Method

### Participants and procedures

The present study represents a secondary analysis of data from the binge‐eating disorder in adolescents (BEDA) study (German Clinical Trials Register: DRKS00000542), a randomized controlled trial comparing individual CBT to waitlist control for adolescents with BED (Hilbert, 2013; Hilbert et al., 2020). The Ethical Committee of Leipzig University (235‐10‐23082010) approved all study procedures. All adolescents and at least one parent (for adolescents aged <18 years) completed informed consent or assent, respectively, prior to initiating any study procedures. Recruitment occurred between April 2012 and August 2014 and employed a population‐based, school‐based, and clinical strategy to engage families. To be included, adolescents had to be between the ages of 12–20 years, and meet the criteria for DSM‐5 or ICD‐11 BED (American Psychiatric Association, 2013; World Health Organization, 2019). Exclusion criteria were: current diagnosis of an eating disorder other than BED (lifetime history of anorexia nervosa or bulimia nervosa was not exclusionary, but none of the adolescents enrolled reported a history of either diagnosis); a current psychotic or bipolar disorder, reported substance misuse, or suicidal ideation (assessed during phone screening using items from the Diagnostic Interview for the Assessment of Mental Disorders in Children and Adolescents; Schneider, Pflug, In‐Albon, & Margraf, 2017); serious unstable medical problems; usage of antipsychotic or weight‐affecting medication [including stimulant medications for attention deficit‐hyperactivity disorder (ADHD)]; concurrent participation in psychotherapy, inpatient treatment, or behavioral weight loss treatment; current pregnancy or lactation; and inability to comply with study procedures.

After a baseline visit including written informed assent and consent, eligibility confirmation, and measurement of height, weight, eating‐related psychopathology, and neurocognitive functioning, adolescents were randomly assigned to either CBT (*n* = 37) or waitlist control (*n* = 36). In this analysis, pre‐ and posttreatment data from the CBT and waitlist group were pooled such that all participants in the current analysis had received CBT by posttreatment and all subsequent time points. Of note, participants assigned to CBT did not differ from those assigned to the waitlist on any baseline demographic or anthropometric characteristics, LOC eating frequency, or any measures of executive functioning performance (all *p*s > .34).

CBT was manualized (Hilbert & Tuschen‐Caffier, 2010) and delivered in 20 individual, face‐to‐face 50‐min sessions with a therapist over 4 months, with high degrees of treatment fidelity (Puls, Schmidt, & Hilbert, 2019). Two sessions per week were scheduled for the first month of treatment (sessions 1–8), and one session per week was scheduled for months 2–4 (sessions 9–20). Consistent with prior data suggesting the efficacy of adolescent‐focused treatment for BED symptomatology with limited or no parental involvement (e.g. DeBar et al., 2013), family sessions were not a routine part of adolescent‐focused CBT and were arranged only when necessary. However, parents received standardized information letters each month about BED, general intervention foci, and recommendations for daily routines. Participants were not given scheduling reminders prior to therapy sessions.

Similar to adult CBT for BED, treatment included an initial phase focused on enhancing motivation; an intensive phase with modules on establishing regular eating patterns, addressing maladaptive cognitions regarding shape and weight, and managing stress; and a final phase focused on preventing relapse. Developmental adaptations included an enhanced number of motivational practices with a lower level of complexity; decreased focus on cognitive interventions (to simplify and concretize the intervention as much as possible for adolescents as young as age 12); and consideration of adolescent‐specific maintenance factors (e.g. identity development, family functioning). A total of *n* = 5 patients (6.8%) terminated therapy prematurely. Follow‐up visits occurred at 6‐, 12‐, and 24‐months posttreatment.

### Measures

#### Demographic variables and covariates

Participants self‐reported their *age*, *gender*, and *nationality*, while parents reported their education, household income and occupational status to generate the Winkler Index of *socioeconomic status* (SES; Winkler & Stolzenberg, 1999). Race and ethnicity were not assessed given the relative homogeneity of the population in Leipzig (<5% non‐German according to 2020 census data) and German norms related to recording of these data in academia (i.e. historical persecution of individuals based on racial and ethnic background in Germany, which has led to cultural taboos around collecting and protecting such data; Boytchev, 2023). A composite of the vocabulary and matrix reasoning subtests of the Wechsler Intelligence Scales (<16 year: Petermann & Petermann, 2008; ≥16 year: von Aster, 2006) were used to determine *general intellectual functioning*.

#### Eating and weight outcomes

The German version of the *Eating Disorder Examination* (EDE; Fairburn, 2008; Hilbert & Tuschen‐Caffier, 2016) was administered by trained interviewers masked to condition. The EDE is a semi‐structured interview used to diagnose BED according to the DSM‐5 (American Psychiatric Association, 2013), and to assess the frequency of LOC eating episodes over the 3 months prior to assessment. A subset of audio‐recorded baseline EDEs (26%) were coded by an independent rater to derive interrater reliability for objective and subjective binge eating frequency (intraclass correlation coefficients = .96 and 1.00, respectively). Body weight and height were measured objectively using calibrated instruments to calculate *body mass index* (BMI; kg/m^2^), and converted to standardized (zBMI) scores for age and sex based on a pooled reference population of more than 34,000 children from different regions in Germany (Kromeyer‐Hauschild et al., 2001).

#### Executive functioning predictors

The paper‐and‐pencil Stroop Color‐Word Interference Test (CWIT; Bäumler & Stroop, 1985), which has strong convergent and divergent validity (Bäumler & Stroop, 1985) and showed high internal consistency in this sample (α = .98), was used as a measure of *inhibition*. Participants first read aloud color names printed in black ink, next named the color of a series of colored bars, and finally named the color of the ink in which a color word was printed rather than the word itself. Longer response time (reflected in *T*‐scores) for the final (color–word interference) trial indicate poorer inhibition. A paper‐and‐pencil D2 Concentration Endurance Test (D2; Brickenkamp, 1962; so named after the target symbol letter ‘d’ as described below), which has good convergent and divergent validity (Bates & Lemay, 2004; Eser, 1987), was administered to assess sustained attention. Adolescents visually scanned 14 lines of stimuli in 20 s to detect target symbols (letter ‘d’) among distractors. Correct responses minus errors (omission of target symbols or striking out distractor symbols) was used to calculate a *concentration performance* score, with higher scores indicating better performance. The Comprehensive Trail Making Test (CTMT; Reynolds, 2002), which has good convergent and divergent validity (Reynolds, 2002; Smith et al., 2008) and demonstrated high internal consistency in the current sample (*r* = .91), was administered to measure *attention* and *cognitive flexibility*. The first three trials, which measure attention, require connection of numbers surrounded by distractors in an ascending order (simple sequencing; SS). The last two trials, which measure cognitive flexibility, require alternation in ascending order between numbers in numeric and word forms and between numbers and letters, respectively (complex sequencing; CS). Higher *T*‐scores reflect better attention and flexibility, respectively. Finally, the Iowa Gambling Task (IGT; Bechara, Damasio, Damasio, & Anderson, 1994), which has good construct validity (Buelow & Suhr, 2009), was used to measure *decision‐making* (Dunn, Dalgleish, & Lawrence, 2006). Participants aimed to maximize financial earnings by making card choices which lead to either variable reward or combined reward and penalty. Net scores were calculated by subtracting the total number of disadvantageous (penalty) choices from the total number of advantageous (reward) choices, with higher scores reflecting more impulsive decision‐making.

Parents completed the Behavior Rating Inventory of Executive Function (BRIEF; Gioia, Isquick, Guy, & Kenworthy, 2000), an 86‐item, psychometrically sound (Jarratt, Riccio, & Siekierski, 2005; McCandless & O' Laughlin, 2007) measure of child *executive function behavior symptoms in daily life*, including inhibition, set‐shifting, and working memory, along with other constructs. Items are scored from ‘1’ (never) to ‘3’ (often) with higher *T*‐scores reflecting poorer functioning. The global executive composite (BRIEF‐GEC), a summary score incorporating all clinical scales, was used in the current study (current α = .89).

### Statistical analysis

All analyses were conducted using Stata. The parent trial was powered (95%) to detect an effect size of *d* = 1.0 for binge‐eating improvement, after accounting for attrition. Given very high correlations among the number of LOC eating episodes reported during months 1, 2, and 3 at all time points (mean *r* = .74), we included the sum of LOC episodes reported across the past 3 months in all analyses to make full use of the data. Although LOC eating episode frequency was highly positively skewed over time, consistent with the main outcomes of the trial (Hilbert et al., 2020), none of the attempted transformations normalized the data, and thus raw LOC frequency values were retained in subsequent analyses. Inspection of plotted data revealed no significant outliers in LOC frequency at any time point. As a first step, bivariate Spearman correlations between the predictor variables (BRIEF‐GEC, CWIT, D2, CTMT‐SS, CTMT‐CS, and IGT), dependent variables (LOC eating frequency, zBMI, number of sessions attended, and attrition rate) and putative covariates (age, gender, SES, and general intellectual functioning) were calculated to address any potential multicollinearity issues in the subsequent analyses. Since none of the predictor or dependent variables were strongly correlated (absolute *r* range = .02–.43; see Table S1), all variables were included in the relevant models. Multiple imputation strategies were considered to minimize missing data; however, to avoid producing biased estimates in light of the sample size, missing data were instead removed from analyses using list‐wise deletion of cases. A total of 291 data points were available for analyses involving LOC eating frequency, and a total of 236 data points were available for analyses involving zBMI. Participants who were and were not included in these full models were compared on sociodemographic characteristics using *t*‐tests and chi‐square analyses.

#### Modeling treatment outcome

Next, to examine effects of baseline executive functioning measures as predictors of treatment outcome, multilevel mixed‐effects linear regression analyses were conducted. Dependent variables included LOC eating frequency (*n* = 203 observations after accounting for missing values over time) and zBMI (*n* = 179 observations after accounting for missing values over time) modeled as repeated measures from baseline through posttreatment and follow‐up. Independent variables included fixed effects of time (baseline; posttreatment; 6‐month follow‐up; 12‐month follow‐up; and 24‐month follow‐up), executive functioning scores (predictors), and covariates of age, gender, SES, and general intellectual functioning. We considered including LOC eating frequency as a covariate in the model predicting zBMI changes, and including zBMI as a covariate in the model predicting changes in LOC eating frequency, but given the small correlation between these two variables (*r* = −.013), we opted to leave these covariates out of the respective models.

#### Modeling treatment engagement

Finally, two separate regression models were used to evaluate the effects of baseline executive functioning measures as predictors of treatment engagement. Linear regression was used to assess the effects of baseline executive functioning (independent variable) on number of sessions attended (dependent variable), adjusting for age, gender, SES, and general intellectual functioning (covariates). Logistic regression was used to examine the effects of baseline executive functioning (independent variable) on treatment attrition (dependent variable), adjusting for age, gender, SES, and general intellectual functioning (covariates).

For all models, only independent variables and/or covariates significant at *p* < .25 were retained for final analyses; results of these final, more parsimonious models are reported henceforth.

## Results

### Descriptive characteristics

Of the 73 participants (including those randomized to CBT and those who were offered CBT after being waitlisted), 87.7% (*n* = 64) initiated treatment and 68.5% completed at least 10/20 sessions (*n* = 50). A total of 56 (76.7%) adolescents completed posttreatment assessments, and *n* = 54 (73.9%), *n* = 56 (76.7%), and *n* = 50 (68.5%) completed 6‐, 12‐ and 24‐month follow‐up assessments, respectively. Participants who were included in the full regression models examining LOC eating frequency, zBMI, attrition, and attendance (*n* = 47) had significantly higher zBMI (*p* = .039) than those who were not (*n* = 26); they did not differ age, sex, SES, or general intellectual functioning (all *p*s > .26). Please see Hilbert et al. (2020) for the main outcomes of the trial.

Participants were mostly female (82.2%; *n* = 60), aged 15.0 years, on average (*SD* = 2.5), and of German nationality (87.7%; *n* = 64), with an average SES score of 11.9 (*SD* = 4.0), corresponding to the middle class. Most adolescents had overweight/obesity (zBMI ≥1.3; 75.3%; *n* = 55), with an average zBMI of 1.9 (*SD* = 1.0). See Table 1 for additional descriptive characteristics, including baseline scores on executive functioning measures.

**Table 1:** Descriptive characteristics, *M* (*SD*) unless otherwise indicated (*N* = 73)

Measure	*M* (*SD*)	Range
Sociodemographic features and covariates		
Age, years	15.0 (2.5)	12 to 21
Sex, *n* (% female)	60 (82.2)	–
Winkler index of socioeconomic status	11.9 (4.0)	3 to 21
Nationality, *n* (% German)^a^	64 (87.7)	–
General intellectual functioning	11.9 (2.3)	7.5 to 16.0
Eating‐ and weight‐related outcomes		
Loss of control eating frequency	31.0 (26.7)	2.0 to 154.0
zBMI	1.9 (1.0)	−0.9 to 4.2
Executive functioning predictors		
BRIEF Global Executive Composite, *T*‐score	60.1 (11.7)	39.0 to 85.0
Stroop Color‐Word Interference Test, *T*‐score	37.0 (13.9)	6.0 to 76.0
D2 Concentration Endurance Test, correct response—errors	57.0 (24.6)	7.0 to 98.0
Comprehensive Trail Making Test‐simple sequencing, *T*‐score	50.7 (11.7)	26.3 to 77.3
Comprehensive Trail Making Test‐complex sequencing, *T‐*score	48.8 (9.9)	35.0 to 77.5
Iowa Gambling Task, net score (reward—penalty choices)	−7.4 (28.7)	−88.0 to 64.0

zBMI, age‐ and sex‐adjusted body mass index (kg/m^2^) *z*‐score; BRIEF, Behavior Rating Inventory of Executive Function. Loss of control eating frequency reflects the sum of objectively and subjectively large binge‐eating episodes reported in the 3 months prior to assessment.

^a^ Of the nine participants reporting a non‐German identity, five did not respond to questions on nationality, one self‐identified as Greek, one as Italian, one as Kazhak, and one as Afghan.

### Predictors of CBT outcome

**Figure 1:**
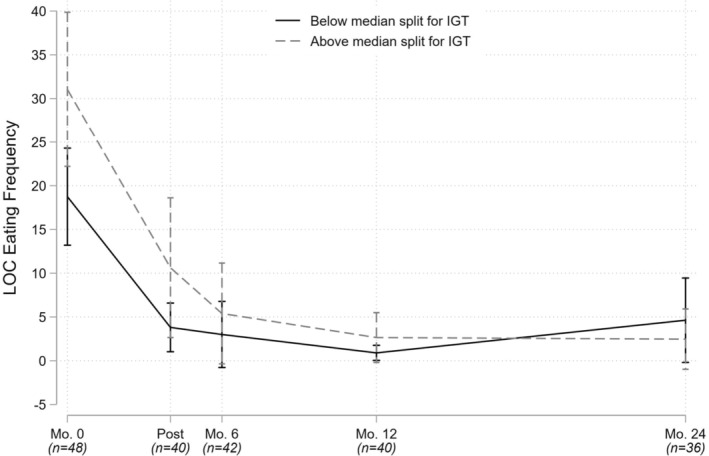
Association between baseline impulsive decision making and changes in loss of control eating frequency during and after cognitive‐behavioral therapy. LOC, loss of control; IGT, Iowa Gambling Task. LOC eating frequency reflects the number of objectively and subjectively large binge‐eating episodes reported in the 3 months prior to assessment. Median splits were used for illustrative purposes only; statistical analyses examined the predictors continuously

**Figure 2:**
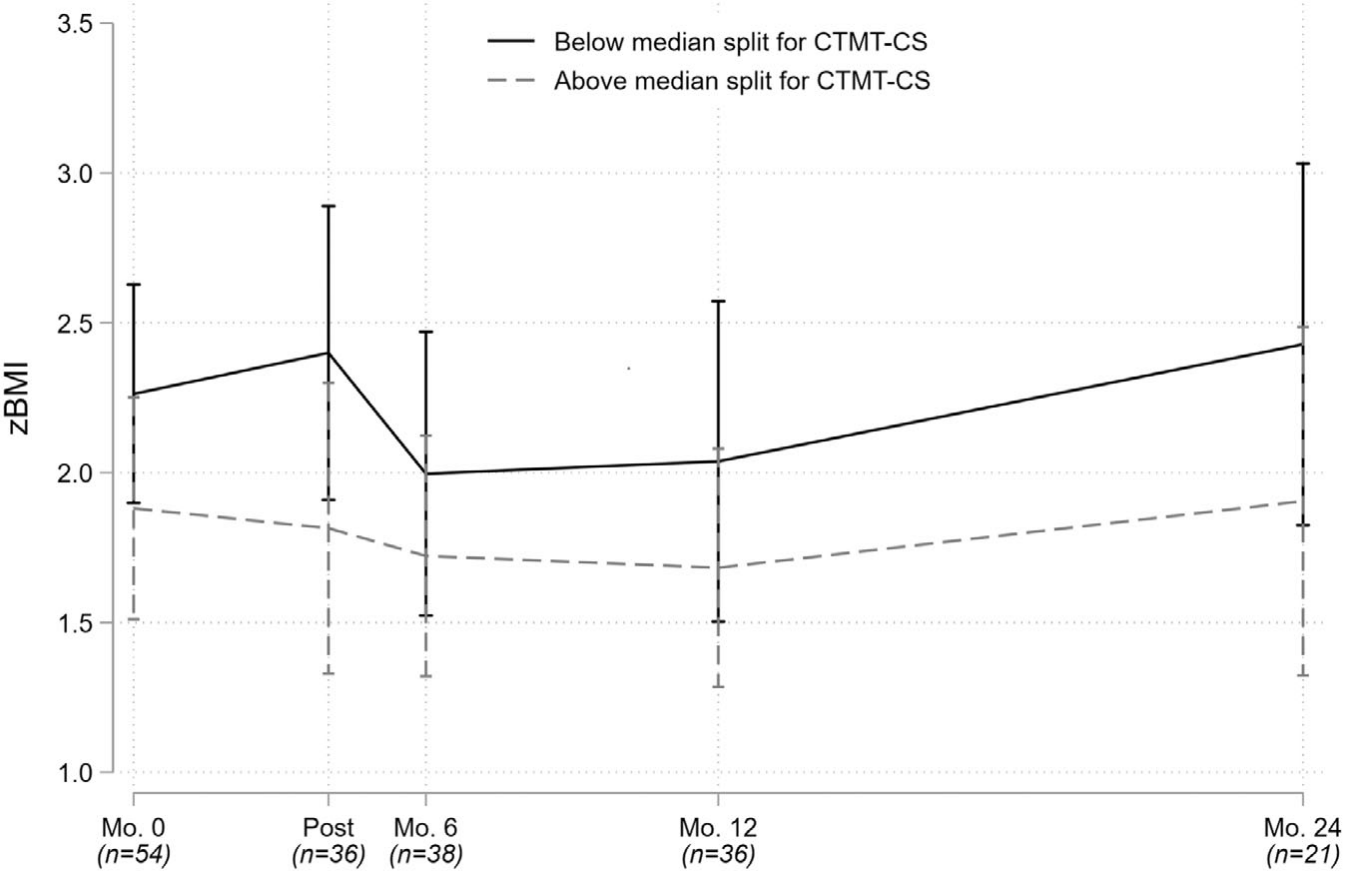
Association between baseline cognitive flexibility and standardized body mass index during and after cognitive‐behavioral therapy. zBMI, standardized body mass index (kg/m^2^); CTMT‐CS, Comprehensive Trail Making Test‐Complex Sequencing index. Median splits were used for illustrative purposes only; statistical analyses examined the predictors continuously

The first set of models examined executive functioning variables as predictors of LOC eating frequency and (separately) zBMI from baseline through 24‐month follow‐up. The former model adjusted for sex and SES, and the latter for age and SES (general intellectual functioning was considered as a covariate, but was dropped in favor of a more parsimonious model including only covariates significant at *p* < .25). We found that more impulsive decision‐making, as reflected in higher baseline scores on the IGT, was prospectively associated with more frequent LOC eating following treatment (*β* = .12; *p* = .017), while poorer cognitive flexibility, as reflected in lower *T*‐scores on the CTMT‐CS, was prospectively associated with higher zBMI following treatment (*β* = −.03; *p* = .003; see Table 2; Figures 1 and 2).

**Table 2:** Regression models predicting cognitive‐behavioral therapy attendance and outcome through 24‐months posttreatment

	LOC eating frequency						zBMI						Attendance					
	Adjusted model (*n* = 193 observations from 47 participants)			Final model (*n* = 203 observations from 49 participants)			Adjusted model (*n* = 162 observations from 47 participants)			Final model (*n* = 179 observations from 52 participants)			Adjusted model (*n* = 47)			Final model (*n* = 51)		
Predictor	β	CI	*p*	β	CI	*p*	β	CI	*p*	β	CI	*p*	β	CI	*p*	β	CI	*p*
BRIEF‐GEC	−.05	−0.22, 0.13	.60	–	–	–	.00	−0.02, 0.02	.99	–	–	–	−.06	−0.25, 0.14	.57	–	–	–
CWIT	−.11	−0.30, 0.08	.24	−.15	−0.33, 0.04	.12	−.01	−0.03, 0.02	.48	–	–	–	.00	−0.18, 0.19	.98	–	–	–
D2	−.14	−0.32, 0.03	.11	−.10	−0.25, 0.05	.20	.00	−0.01, 0.02	.70	–	–	–	.00	−0.12, 0.12	.96	–	–	–
CTMT‐SS	.03	−0.21, 0.28	.79	–	–	–	−.01	−0.04, 0.02	.43	–	–	–	−.03	−0.28, 0.22	.81	–	–	–
CTMT‐CS	.06	−0.25, 0.37	.72	–	–	–	−.02	−0.05, 0.01	.11	**−.03**	−0.05, −0.01	**.003**	−.09	−0.39, 0.21	.54	–	–	–
IGT	.10	0.00, 0.20	.04	**.12**	0.02, 0.21	**.02**	.00	−0.01, 0.01	.47	–	–	–	.07	0.00, 0.14	.06	**.07**	0.01, 0.14	**.02**

LOC, loss of control; zBMI, age‐ and sex‐adjusted body mass index (kg/m^2^) *z*‐score; CI, 95% confidence interval; BRIEF‐GEC, Behavior Rating Inventory of Executive Function Global Executive Composite (*T*‐score); CWIT, Stroop Color‐Word Interference Test (*T*‐score); D2, D2 Concentration Endurance Test (correct response—errors); CTMT‐SS, Comprehensive Trail Making Test‐simple sequencing (*T*‐score); CTMT‐CS, Comprehensive Trail Making Test‐complex sequencing (*T*‐score); IGT, Iowa Gambling Task (net score: reward—penalty choices). Adjusted models included age, sex, and socioeconomic status as covariates. Final models included only variables significant at *p* < .25 in the adjusted models. LOC eating frequency reflects the sum of objectively and subjectively large binge‐eating episodes reported in the 3 months prior to assessment. Bolded values indicate statistically significant at *p* < .05 results in the final parsimonious models.

### Predictors of CBT engagement

The second set of models examined executive functioning predictors of session attendance and attrition (age, sex, SES, and general intellectual functioning were considered as covariates, but were dropped in favor of a more parsimonious model as none of these covariates were significant at *p* < .25). More impulsive decision‐making, as reflected in higher baseline scores on the IGT, was prospectively associated with a greater number of sessions attended (*β* = .07; *p* = .019). None of the executive functioning variables predicted attrition (all *p*s > .12; see Table 3).

**Table 3:** Logistic regression model predicting cognitive‐behavioral therapy attrition

Predictor	Adjusted model (*n* = 47)			Final model (*n* = 51)		
	*O.R*.	CI	*p*	*O.R*.	CI	*p*
BRIEF global executive composite, *T*‐score	0.96	0.89, 1.04	.34	–	–	–
Stroop color‐word interference test, *T*‐score	1.03	0.97, 1.10	.34	–	–	–
D2 concentration endurance test, correct response—errors	1.00	0.96, 1.04	.82	–	–	–
Comprehensive trail making test‐simple sequencing, *T*‐score	1.00	0.90, 1.11	.95	–	–	–
Comprehensive trail making test‐complex sequencing, *T‐*score	0.93	0.85, 1.02	.13	0.96	0.90, 1.03	.30
Iowa gambling task, net score (reward—penalty choices)	1.02	0.99, 1.06	.12	1.02	1.00, 1.05	.08

O.R., odds ratio; CI, 95% confidence interval; BRIEF, Behavior Rating Inventory of Executive Function. Adjusted models included age, sex, and socioeconomic status as covariates. Final model included only variables significant at *p* < .25 in the adjusted model.

## Discussion

The current study aimed to investigate the impact of executive functioning on outcome and engagement in CBT for adolescents with BED. In partial support of our hypotheses, we found that more impulsive decision‐making prior to treatment predicted more frequent LOC eating following CBT (despite also predicting higher session attendance), and poorer cognitive flexibility prior to treatment predicted higher zBMI following treatment, although effects were generally small and further exploration of their clinical significance is needed. In addition, results should be interpreted with caution in light of the exclusion of individuals with missing data, which could have affected generalizability, and the waitlist control design, which could have resulted in changes in executive functioning between the time of assessment and the start of treatment (although of note, only 4 months passed between the baseline evaluation and the start of treatment for waitlisted participants, meaning that any changes in executive functioning were likely modest).

Given that CBT is a present‐focused, solution‐oriented treatment with an expectation that homework be undertaken outside of therapy sessions (e.g. stimulus control, exposure exercises; Fairburn, 2008; Hilbert & Tuschen‐Caffier, 2010), we speculate that relative decrements in decision‐making and cognitive flexibility may make it more challenging for youth to recall and practice newly learned skills in real‐world contexts and settings, especially those in which there are increased demands on executive functioning capacity (e.g. during high‐risk situations in which vulnerability to dysregulated eating is increased, such as in the presence of typical ‘binge’ foods or at times of elevated distress). Augmenting CBT with executive function training targeting decision‐making and cognitive flexibility (Hilbert et al., 2021), and/or delivering it in real time across naturalistic settings (e.g. through mHealth interventions), may represent promising ways to strengthen effects on eating and weight outcomes in adolescents with BED, especially those with poorer executive functioning.

CBT involves a strong focus on building skills related to pausing and evaluating contextual triggers to problematic behaviors, and identifying more adaptive ways to respond. As such, it is perhaps not surprising that youth who struggle with impulsive decision‐making may find it more challenging to implement these foundational skills in the context of events and emotions that precipitate risk for LOC eating [while at the same time, attending more sessions (consistent with an earlier study examining attrition from a digital health intervention for adolescents with obesity; Desmet, Fillon, Thivel, Tanghe, & Braet, 2023), perhaps reflecting increased awareness of a need for support in managing their eating behavior]. Just‐in‐time adaptive interventions (JITAIs), which use idiographic data to develop decision rules about when and where to intervene and then deliver real‐time interventions in the moments/contexts they are most needed (Spruijt‐Metz & Nilsen, 2014), may be particularly relevant to these youth. Such interventions have been proposed for the implementation of CBT for adults (although efficacy is unclear; Juarascio, Presseller, Srivastava, Manasse, & Forman, 2023), but have yet to be applied in an adolescent population.

Although cognitive flexibility was not associated with CBT outcomes as reflected in LOC eating outcomes, it was associated with poorer weight outcomes over time. Cognitive flexibility is an executive function that involves rapidly adjusting one's cognitions, attitudes, and/or behaviors to align with contextual changes (e.g. environment, task goals; Diamond, 2013). It is possible that cognitive inflexibility influences other obesogenic eating behaviors, aside from LOC eating, that mechanistically affect weight regulation. For example, adolescents who struggle with cognitive inflexibility may have a difficult time generating alternative responses to novel social situations involving food, increasing vulnerability to engage in maladaptive eating behaviors that do not involve LOC (e.g. eating in the absence of hunger; Schmidt et al., 2023). This and other possibilities should be explored in future research.

The current study was marked by several strengths, including the assessment of a range of executive functioning constructs using both behavioral tasks and parent‐report measures (although all measures were administered in oral or written format, which may introduce some variability in scoring); examination of both treatment engagement *and* outcome (although, of note, engagement was conceptualized as session attendance and treatment completion; measures of compliance such as homework completion were not available); the availability of long‐term follow‐up data; the rigorous assessment of BED and related features; and the inclusion of both male‐ and female‐identifying participants.

Despite these strengths, there were several limitations that should be considered in interpreting the data. First, executive functioning was estimated according to performance on general measures, precluding examination of whether food‐specific inefficiencies may affect CBT outcome; was not assessed in parents, who may play an active role in structuring the home food environment (e.g. Pearson, Biddle, & Gorely, 2009), are crucial supports around developmental needs of their children (including fostering time management skills, encouraging adherence to treatment goals, and transporting youth to therapy sessions; Smetana & Rote, 2019), and may contribute (through genes and/or modeling) to their children's executive functioning and eating‐related psychopathology (Barakat et al., 2023; Halse, Steinsbekk, Hammar, Belsky, & Wichstrøm, 2019); and was measured in youth at baseline only, precluding examination of how *changes* in executive functioning may have related to differences in eating and weight outcomes over time. Indeed, prior studies have shown that improvements in executive functioning during the course of CBT are associated with better CBT outcome in pediatric anxiety (Godovich et al., 2020); if similar improvements occur in the context of CBT for BED, it could explain why many of the executive functioning domains that were hypothesized to predict poorer treatment outcome (e.g. inhibition, attention) were not associated with eating‐ or weight‐related changes following CBT. Second, although youth with ADHD were included in the sample (an important strength given the well‐documented association between ADHD and binge eating in youth; Villa et al., 2023), stimulant medication usage was exclusionary (and resulted in a total of three adolescents being excluded from the sample), which may both limit generalizability, while also limiting a confounding factor that could bias executive functioning performance. An additional limitation related to generalizability was the demographically homogeneous sample, and the exclusion of individuals with missing data, which necessitate replication in larger and more diverse samples. Indeed, although the parent trial was sufficiently powered to identify differences in outcomes by treatment assignment, the sample size was modest for many of these secondary analyses, which may have limited power to identify effects of executive functioning on LOC eating and weight outcomes (and may have also contributed to non‐normal distributions for some of the models, although it should be noted that mixed‐effects models have been found to be robust to violations of normality assumptions; Schielzeth et al., 2020). Finally, as this was a secondary analysis of an efficacy trial designed to evaluate CBT versus waitlist control, the study lacked a suitable control condition (i.e. waitlisted participants received CBT after 4 months, rendering longer‐term outcome data incomparable across conditions), limiting ability to definitively establish whether executive functioning constructs *moderate* treatment outcome or assess whether change in the outcomes was due to CBT versus regression to the mean.

Although accumulating research suggests that executive functioning may play a role in the onset and/or maintenance of dysregulated eating and excess weight status in youth (Lavagnino et al., 2016), this was the first study, to our knowledge, to investigate effects on CBT outcome for adolescents with BED (and one of very few studies to investigate its effects on CBT outcome for other mental health conditions in youth, with prior work focusing exclusively on anxiety‐spectrum disorders; Godovich et al., 2020; Hybel et al., 2017; McNamara et al., 2014). Our findings suggest several directions for future research, including the possibility of augmenting CBT with a focus on executive functioning skill building and practice for youth with BED and lower pre‐treatment executive functioning, and/or delivering it in real‐time, real‐world contexts via digital modalities. Further research is also required to understand whether youth with executive dysfunctions may benefit from treatment approaches that are not as strongly predicated on the presence of robust executive functioning skills (e.g. relying more heavily on parental support to offset youth executive functioning deficiencies). Finally, while replication is clearly needed, assessing executive functioning prior to initiating care for adolescent BED may be warranted to better understand prognosis and/or potentially to identify those who may benefit from augmented treatment. An approach that accounts for time‐ and resource‐related constraints involved in administration (e.g. identifying one or two rapidly administered and cost‐effective measures of constructs with the most robust impacts on treatment outcome) should be considered to optimally support feasibility and scale.

**Key points**

**What's known**

- Adolescents with loss of control (LOC) eating often struggle with diminished executive functioning relative to their peers.
- Executive functioning deficits may undermine treatment outcomes for other mental health conditions.

**What's new**

- Impulsive decision‐making predicted more frequent LOC eating following treatment and better session attendance.
- Lower cognitive flexibility predicted poorer weight outcomes following treatment.

**What's relevant**

- Strengthening executive functioning could improve eating and weight disorder outcomes among adolescents with binge‐eating disorder who have lower pre‐treatment executive functioning.

## Supporting information


**Table S1.** Correlation matrix of associations among executive functioning constructs.

## Data Availability

Data are available upon reasonable request from senior author Anja Hilbert (Anja.Hilbert@medizin.uni‐leipzig.de).
