# Supplementary material for: Executive functioning and treatment outcome among adolescents undergoing cognitive‐behavioral therapy for binge‐eating disorder
Source: J Child Psychol Psychiatry. 2024 Jun 28;66(1):64–74. doi: 10.1111/jcpp.14031 (PMC11652412; doi:10.1111/jcpp.14031)
Supplement: Supplementary file 1 — Table S1. Correlation matrix of associations among executive functioning constructs. [file JCPP-66-64-s001.docx]

| **Table S1.** Correlation matrix of associations among executive functioning constructs. | | | | | | |
| --- | --- | --- | --- | --- | --- | --- |
|  | 1 | 2 | 3 | 4 | 5 | 6 |
| 1. BRIEF Global Executive Composite | --- | -.36 | -.41 | -.42 | -.40 | -.16 |
| 2. Stroop Color-Word Interference Test | --- | --- | .36 | .36 | .48 | .10 |
| 3. D2 Concentration Endurance Test | --- | --- | --- | .51 | .57 | .16 |
| 4. Comprehensive Trail Making Test-simple sequencing | --- | --- | --- | --- | .55 | .12 |
| 5. Comprehensive Trail Making Test-complex sequencing | --- | --- | --- | --- | --- | .27 |
| 6. Iowa Gambling Task | --- | --- | --- | --- | --- | --- |
| *Note:* BRIEF=Behavior Rating Inventory of Executive Function | | | | | | |
